# Matricellular proteins: multifaceted extracellular regulators in tumor dormancy

**DOI:** 10.1007/s13238-014-0023-6

**Published:** 2014-02-22

**Authors:** Tiantian Wu, Gaoliang Ouyang

**Affiliations:** State Key Laboratory of Cellular Stress Biology, Innovation Center for Cell Biology, School of Life Sciences, Xiamen University, Xiamen, 361102 China

Tightly controlled extracellular matrix (ECM) homeostasis and remodeling is critical for normal organ homeostasis, wound healing and tissue repair. However, excessive or uncontrolled ECM deposition contributes to aberrant homeostasis of tissue microenvironment in various inflammatory diseases and tumors. Matricellular proteins are a set of structurally unrelated ECM proteins that do not exert a primary role in tissue architecture but have regulatory roles in embryonic development, tissue injury, inflammation and tumor progression. Two recent studies demonstrated that matricellular proteins in the ECM surrounding dormant tumor cells may determine the fate of tumor cells to remain quiescent or undergo metastatic outgrowth. The identification of matricellular proteins in regulating ECM homeostasis and remodeling specific organ niches during tumor dormancy may provide potential novel extracellular targets for the development of therapeutic interventions against tumor dormancy.

**Figure 1:**
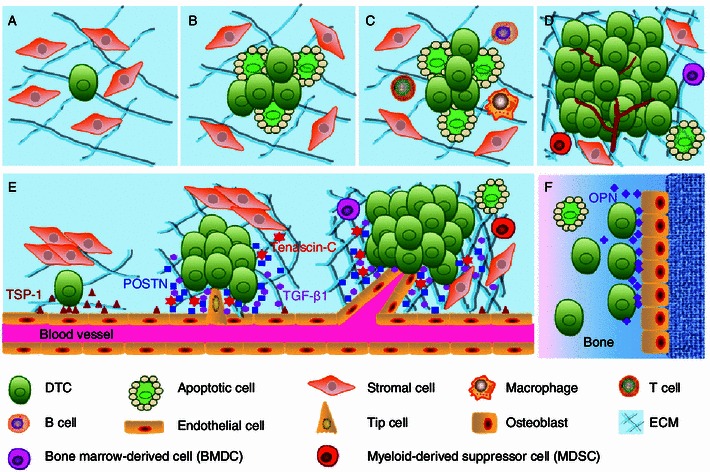
**Tumor dormancy and matricellular proteins**. Three types of tumor dormancy exist: cellular dormancy (A), angiogenic dormancy (B) and immunological dormancy (C). Cancer cells enter into G_0_–G_1_ arrest during cellular dormancy. Angiogenic dormancy is regarded as a state during which pro-angiogenic factors are balanced with anti-angiogenic factors, resulting in poor vascularization and limited tumor size. Immunological dormancy refers to a state during which the active immune system clears most of tumor cells, leading to an equilibrium between cell population and immunological clearance. (D) DTCs exit from tumor dormancy and turn into metastatic outgrowths. (E) TSP-1 is rich in mature vessel stalks and maintains disseminated breast cancer cells in a dormant state, whereas POSTN, tenascin-C and TGF-β1 are distributed at neovascular tips and enable DTCs to escape from the dormant state and undergo metastatic outgrowth. (F) OPN assists acute lymphoblastic leukemia cells in anchoring to the bone marrow endosteal niche and promotes dormancy

Many cancer patients who have undergone surgical resection of their primary tumors suffer from metastatic relapse several months, years or even decades later. Current evidence supports the idea that tumor cells can be disseminated at an early stage of tumor progression. The disseminated tumor cells (DTCs) exist in a quiescent state at a hostile site, but may switch from quiescence to proliferative metastatic growth in a permissive and supportive microenvironment. Increasing data suggest that tumor dormancy can be regarded as a protracted asymptomatic stage during which tumor cells either remain in a quiescent state or their proliferation is balanced by cell death due to immunosurveillance or insufficient angiogenesis (Aguirre-Ghiso, 2007; Giancotti, 2013; Hensel et al., 2013) (Fig. 1). Similar to other processes in tumorigenesis and progression, tumor dormancy is governed by both intracellular signaling pathways and tissue microenvironment cues. However, how DTCs enter into quiescence and subsequently reactivate, as well as which types of microenvironmental cues contribute to the modulation of tumor dormancy, remains largely unknown. Recently, two intriguing studies indicated that matricellular proteins play a critical role in regulating the quiescent state and enabling dormant DTCs to undergo metastatic growth (Boyerinas et al., 2013; Ghajar et al., 2013).

Ghajar et al. reported that there are two distinct sub-vascular niches when DTCs extravasate at the target metastatic sites. The mature microvascular niche provides suppressive cues to inhibit cell proliferation and induces the sustained quiescence of DTCs, whereas the sprouting neovasculature niche provides stimuli that activate DTCs and promote micrometastatic outgrowth. Among these dormancy-promoting and -inhibiting niches, several matricellular proteins, including thrombospondin-1 (TSP-1) and periostin (POSTN), play crucial roles in regulating DTC dormancy. TSP-1 is enriched in the basement membrane of mature microvessel stalks and maintains DTCs in a dormant state; however, TSP-1 levels are markedly decreased at the sprouting neovascular tips of newly forming vessels. Interestingly, they also found an increased distribution of matricellular proteins, such as POSTN, tenascin-C and SPARC, together with fibronectin and TGF-β1, around these growing tips. The authors further provided evidence that high POSTN and active TGF-β1 expression surrounding neovascular tip cells enables DTCs to exit from dormancy and triggers metastatic relapse (Ghajar et al., 2013) (Fig. 1). At nearly the same time, Boyerinas and colleagues demonstrated that osteoblast-derived osteopontin (OPN) assists acute lymphoblastic leukemia (ALL) cells in anchoring to the bone marrow endosteal niche and promotes tumor dormancy (Fig. 1). OPN neutralization increases leukemia burden by decreasing dormant ALL cells in mice, but reduces minimal residual disease in leukemic mice when synergizes with Ara-C-based chemotherapy (Boyerinas et al., 2013). These results suggest that matricellular proteins TSP-1, POSTN, tenascin-C, SPARC and OPN exhibit critical roles in regulating tumor dormancy.

Two questions arising from these observations are why do so many matricellular proteins actively contribute to the regulation of tumor dormancy, and where do they exert location-specific effects on dormancy? Matricellular proteins are a set of non-structural extracellular matrix (ECM) proteins, mainly including thrombospondins (TSPs), OPN, tenascins, SPARC, POSTN and CCNs, which do not exert a primary role in tissue architecture but regulate similar biological functions in response to external stimuli during embryonic development, tissue injury, inflammation and tumor progression (Bornstein, 1995). TSP-1 is a potent inhibitor of neovascularization and tumorigenesis (Isenberg et al., 2009), whereas POSTN acts as a potent pro-angiogenic factor by binding to integrins to activate PI3K/Akt and FAK signaling pathways, thereby promoting cell survival and angiogenesis (Bao et al., 2004; Shao et al., 2004). Tenascin-C, SPARC, OPN and CCNs are also actively involved in modulating angiogenesis (Chiodoni et al., 2010). Current evidence demonstrates that tumor blood vessels are important routes for DTCs to metastasize to distant sites and are indispensable for tumor development (Liu and Ouyang, 2013). Perivascular niche has been shown to be a critical local microenvironment to regulate self-renewal of cancer stem cells (CSCs) (Calabrese et al., 2007). Interestingly, tenascin-C and POSTN are important regulators of CSC stemness in metastatic niches (Oskarsson et al., 2011; Ouyang et al., 2010; Malanchi et al., 2012; Wang and Ouyang, 2012; Wang et al., 2013). Thus, these matricellular proteins may be key components in perivascular niches and contribute to the maintenance or inhibition of dormancy by regulating endothelial cell proliferation, angiogenic switch and DTC self-renewal.

In bone marrow, the endosteal niche is important for hematopoietic stem cells (HSCs) to maintain quiescence, whereas the perivascular niche accommodates more activated HSCs. Quiescent HSCs reside preferentially at the endosteal region where bone-lining osteoblasts and their secreted proteins are critical components of HSC niches. Interestingly, bone marrow microenvironment is also a critical reservoir for DTCs. When metastasizing to the bone, DTCs take shelter in HSC niches in bone marrow and adopt similar mechanisms to those used by HSCs for homing to bone marrow. That is to say, DTCs may occupy and remodel preexisting physiological HSC niches to help them survive and subsequently grow and metastasize (Wan et al., 2013). OPN has been shown to be a critical component of the HSC niche and to negatively regulate HSC numbers (Nilsson et al., 2005). POSTN, SPARC, tenascin-C, TSP-1 and CCNs can also be secreted by several bone marrow-derived cells, and their aberrant expression is related to DTC bone metastasis. Administration of anti-POSTN antibody or knockdown of tenascin-C inhibits bone metastasis of breast cancer in mice. Thus, the detailed functions of OPN in HSC niches and ALL cell dormancy indicate that other matricellular proteins may also contribute to bone marrow niches and DTC dormancy in bone. Whether these matricellular proteins regulate tumor dormancy in bone marrow HSC niches, perivascular locations and other specific metastatic niches deserves systemic investigation.

How do matricellular proteins maintain dormant tumor cells in a quiescent state or reactivate them to metastatic outgrowth? As a class of structurally unrelated ECM proteins, matricellular proteins serve as links between the ECM and cells to regulate cell-cell and cell-matrix interactions, and their functions are highly dependent upon the cues from local microenvironment. These matricellular proteins are often needed for ECM remodeling during embryonic development and their expressions are highly induced at sites of injury, inflammation or tumors within the adult organism. An excessive or uncontrolled matricellular proteins’ function contributes to aberrant remodeling and homeostasis of tissue microenvironment in various inflammatory diseases and tumors. Matricellular protein knockout mice are often viable, but typically exhibit abnormal responses to mechanical stress, wound healing, inflammation and tumor metastasis. POSTN, for example, can directly interact with collagen I, fibronectin and Notch1 via its EMI domain and interact with tenascin-C and BMP-1 via its FAS1 domains (Norris et al., 2007; Maruhashi et al., 2010; Kii et al., 2010; Tanabe et al., 2010). POSTN and lysyl oxidase (LOX) precursor bind to fibronectin, stabilizing LOX precursor and allowing it to be proteolyzed into mature LOX. Thus, POSTN can recruit BMP-1 onto the fibronectin matrix to promote LOX activity for collagen cross-linking (Kudo, 2011; Conway et al., 2014). This scaffold and remodeling function of POSTN may help us to understand its mechanism in regulating inflammation and tumor metastasis. For example, POSTN is highly expressed in various inflammatory tissues and regulates TGF-β signaling to modulate Th2 inflammatory response (Masuoka et al., 2012; Conway et al., 2014). POSTN is also a critical component in the metastatic niche that promotes DTC metastatic outgrowth (Bao et al., 2004; Ruan et al., 2009; Malanchi et al., 2012; Wang and Ouyang, 2012; Wang et al., 2013). Moreover, POSTN can promote DTC self-renewal and metastatic formation by augmenting Wnt signaling pathway (Malanchi et al., 2012). Interestingly, TSP-1 (Isenberg et al., 2009), tenascin-C (Oskarsson et al., 2011), SPARC (Rivera et al., 2011), OPN (Song et al., 2009) and CCNs (Jun and Lau, 2011) are also actively involved in ECM remodeling and regulating cell survival- and proliferation-related signaling pathways in inflammatory diseases and tumors. Therefore, the prominent roles of these matricellular proteins in inflammatory responses, tumor dormancy and metastasis are to remodel the biophysical and biochemical properties of tissue microenvironment; recruit Wnt, Notch, TGF-β or other ligands to modulate their signaling; and bind with their cell surface receptors to regulate survival- and proliferation-related pathways inside the cells by interacting with growth factors, proteases, cell surface receptors and other structural matrix proteins, as well as themselves. A more detailed understanding of the functions of matricellular proteins in inflammatory and metastatic microenvironments will help to elucidate the role of matricellular proteins in regulating tumor dormancy.

The work by Ghajar et al. and Boyerinas et al. greatly expanded our understanding of the roles of tissue microenvironmental cues in regulating tumor dormancy and subsequent metastatic growth. The identification of matricellular proteins in regulating ECM homeostasis and remodeling specific organ niches during tumor dormancy provides useful extracellular targets for the development of therapeutic interventions to induce or maintain DTCs in a dormant state, or alternatively to activate and then eradicate dormant DTCs.
